# HHS/CDC Legal Response to SARS Outbreak

**DOI:** 10.3201/eid1002.030721

**Published:** 2004-02

**Authors:** James J. Misrahi, Joseph A. Foster, Frederic E. Shaw, Martin S. Cetron

**Affiliations:** * Centers for Disease Control and Prevention, Atlanta, Georgia, USA

**Keywords:** law, legal aspects, lawyers, constitutional law, quarantine, patient isolation

## Abstract

Before the severe acute respiratory syndrome (SARS) outbreak, the Centers for Disease Control and Prevention’s (CDC) legal authority to apprehend, detain, or conditionally release persons was limited to seven listed diseases, not including SARS, and could only be changed using a two-step process: 1) executive order of the President of the United States on recommendation by the Secretary, U.S. Department of Health and Human Services (HHS), and 2) amendment to CDC quarantine regulations (42 CFR Parts 70 and 71). In April 2003, in response to the SARS outbreak, the federal executive branch acted rapidly to add SARS to the list of quarantinable communicable diseases. At the same time, HHS amended the regulations to streamline the process of adding future emerging infectious diseases. Since the emergence of SARS, CDC has increased legal preparedness for future public health emergencies by establishing a multistate teleconference program for public health lawyers and a Web-based clearinghouse of legal documents.

Under our American constitutional structure, the “police power” (the authority of sovereign governments to enact laws and promote regulations that safeguard the health, safety, and welfare of its citizens) is reserved to the states by the 10th Amendment to the U.S. Constitution, while the federal government exercises authority to regulate interstate and foreign commerce (*1*). As a result, state and local health departments have primary responsibility for controlling communicable diseases within their boundaries, while the federal government is primarily responsible for controlling transmission and spread of communicable diseases from abroad and from one state to another. Rapidly spreading epidemic diseases, such as severe acute respiratory syndrome (SARS), have the potential to cross interstate and international borders, potentially overwhelming the ability of any one jurisdiction to respond, despite the appropriate efforts taken by health officials. Recognizing the cross border nature of some communicable diseases and in light of this nation’s constitutional structure, section 361 of the Public Health Service Act (42 United States Code section 264) authorizes the Health and Human Services (HHS) Secretary to make and enforce regulations necessary to prevent the introduction, transmission, and spread of communicable diseases from foreign countries into the United States and from one state or possession into another.

In enacting section 361, Congress recognized “the impossibility of foreseeing what preventive measures may become necessary” (*2*). Accordingly, Congress quite logically delegated to the executive branch the responsibility of designating specific communicable diseases that would be subject to federal isolation and quarantine measures. As enacted in 1944, the statute required the President to list the diseases for which quarantine was authorized through executive order, on recommendation of the HHS secretary and a group known as the National Advisory Health Council (*2*). The first executive order listing “quarantinable” diseases was issued by President Truman on March 26, 1946 (*3*). Presidents Eisenhower, Kennedy, and Reagan issued successive orders in 1954, 1962, and 1983, respectively (*4*–*6*). The quarantinable diseases listed in these executive orders were published in regulations found in 21 Code of Federal Regulations (CFR) Part 1240 and 42 CFR Part 71.

Historically, two sets of regulations promulgating section 361 have existed: one designed to prevent the introduction, transmission, and spread of communicable diseases from foreign countries into the United States and the other designed to prevent the interstate movement of communicable diseases within the United States. The Centers for Disease Control and Prevention (CDC) had administered the foreign quarantine regulations, while the U.S. Food and Drug Administration (FDA) had administered the interstate quarantine regulations. In addition to quarantine, these regulations authorize a variety of other public health measures, including reporting of ill passengers onboard international conveyances, sanitary inspection of arriving vessels and cargo, and restrictions on articles or imports that may be sources of infection to human beings. On August 16, 2000, FDA transferred a portion of its domestic quarantine authority (the portion dealing with persons) to CDC, while retaining its authority to control animals and other products that may transmit or spread communicable diseases (*7*). The portion of FDA’s regulations dealing with persons appearing in 21 CFR Part 1240 was transferred and recodified in CDC’s regulations at 42 CFR Part 70 (*7*). This transfer reduced potential delays in implementing quarantine by consolidating authority to quarantine persons with specified communicable diseases under one federal agency.

As part of its planning for bioterrorism and especially in light of the events of September 11, 2001, HHS sought to further expedite quarantine procedures by reducing potential delays involved in adding new diseases to the list of quarantinable diseases. On June 12, 2002, President Bush signed into law the Public Health Security and Bioterrorism Preparedness and Response Act of 2002, which, among other things, eliminated the need to convene an advisory committee to amend the list of diseases (*8*). The 2002 legislative changes also clarified that federal isolation and quarantine measures apply not just to persons who are infectious but also to persons who have been exposed to a communicable disease and may potentially become infectious (*8*).

## HHS/CDC Legal Response

Before the outbreak of SARS, the list of federal quarantinable diseases in the United States had not been revised since 1983. It included cholera, diphtheria, infectious tuberculosis, plague, smallpox, yellow fever, and viral hemorrhagic fevers such as Marburg, Ebola, and Congo-Crimean (*4*–*6*). Within days of the appearance of SARS, other countries, including Canada, Hong Kong Special Administrative Region, and Singapore instituted restrictive health measures, including large-scale quarantine, to prevent the further spread of the disease. In Ontario, Canada, where SARS-associated coronavirus (SARS-CoV) was transmitting in the population, the provincial government made SARS a reportable, virulent, communicable disease under Ontario’s Health Protection and Promotion Act. This change enabled Ontario public health officers to issue orders to enjoin infected persons from engaging in activities that may transmit SARS. At the federal level, Health Canada also dispatched quarantine officers to international airports in Toronto and Vancouver, screened incoming air passengers from infected areas for SARS, and distributed health alerts at major airports in Canada.

In the United States, the federal executive branch moved rapidly to revise the list of quarantinable communicable diseases by adding SARS to the diseases specified in the April 4, 2003, executive order (*9*). This provided U.S. federal health officials with quarantine powers comparable to those in other countries affected by SARS. Similar to actions taken in other countries, CDC quarantine officers also began screening incoming passengers for symptoms of SARS, distributing health alerts and advisories regarding SARS, and coordinating with airport personnel in the evaluation of sick passengers. Meanwhile, the nature of the disease was rapidly evolving. For example, it was not known whether the name of the disease might change from SARS to something else as more was learned about the disease. To deal with this possibility, the executive order described SARS as follows: “a disease associated with fever and signs and symptoms of pneumonia or other respiratory illness, is transmitted from person to person predominantly by the aerosolized or droplet route, and, if spread in the population, would have severe public health consequences” (*9*). HHS also streamlined the process of adding new quarantinable diseases by eliminating the need to dual-publish the list of diseases in an executive order and in regulations (*10*). Future revisions to the list of quarantinable diseases require only an executive order, which will be posted on the Web at: http://www.cdc.gov and http://www.archives.gov/federal_register (*10*).

CDC has generally deferred to state and local health authorities in the primary use of their own separate “police power” quarantine authorities to restrict the movement of persons within their boundaries. During the SARS outbreak, for example, some states relied on their own legal authorities to control the movement of persons, so it was not necessary for CDC to invoke federal quarantine power to compel the isolation or quarantine of a person within a state. On the basis of a long and successful history of collaboration with the states during public health emergencies, CDC is likely to invoke federal quarantine power only rarely, such as at ports of entry or other time-sensitive situations. In these situations, and in others that are, for example, inherently and necessarily beyond the capacity of state and local jurisdictions to control, CDC has the legal tools it needs to quarantine and isolate persons for SARS and other specified communicable diseases.

## Future Action

While this country was fortunate in that SARS did not reach the scale of the outbreak in Toronto or Singapore, a lesson learned from the outbreak is that federal, state, and local officials will have to work closely in coordinating quarantine actions at all levels of government. Historically, public health legal counsels have served as “technicians” in public health practice, asked by the public health agencies they serve to interpret arcane statutory language and render opinions. Legal preparedness, however, is increasingly being viewed as a critical component of state and local government public health preparedness activities. As demonstrated repeatedly, in the SARS outbreak (quarantine/isolation); in the introduction of monkeypox in the Western Hemisphere (restrictions upon the exotic animal pet trade); and during West Nile virus season (mosquito abatement/spraying programs), legal issues are nearly always intertwined with public health responses. During emergencies, communication among public health lawyers at all levels (federal, state, and local) is a crucial part of the “new normal” in public health. Until recently, however, there was no ready means for public health lawyers to communicate rapidly among themselves and quickly access relevant legal information.

During the SARS outbreak, CDC established a series of telephone conferences, whereby federal, state, and local public health lawyers could discuss important legal issues of the day and trade ideas about pending legal problems. These teleconferences were particularly useful in exchanging information concerning the interplay of quarantine authority at the federal, state, and local levels and discussion of procedural requirements involved in executing isolation or quarantine orders. These legal teleconferences were reinstituted and held daily during the peak of the monkeypox outbreak. Additionally, during the monkeypox outbreak, CDC developed a Web-based clearinghouse where just-issued legal documents such as gubernatorial executive orders and state and local health department rules could be posted. CDC, through its Public Health Law Program, plans to expand the scope of this clearinghouse to reduce the time required to identify relevant legal documents and disseminate them to public health legal counsels on a “real time” basis. The clearinghouse is available at: http://www.phppo.cdc.gov/od/phlp/. The addition of a Web-based clearinghouse and a teleconference capacity increases CDC’s effectiveness in responding to public health emergencies by more fully integrating lawyers into the public health response.
